# Risk-Adapted Selective Elective Nodal Irradiation in the Total Neoadjuvant Therapy Era for Rectal Cancer

**DOI:** 10.3390/medicina62040680

**Published:** 2026-04-02

**Authors:** Seung-Gu Yeo, Min-Jeong Kim, Kwang Hwan Cho, Jina Yun, Dae Ro Lim, Jung Cheol Kuk

**Affiliations:** 1 Department of Radiation Oncology, Soonchunhyang University College of Medicine, Soonchunhyang University Hospital, Bucheon 14584, Republic of Korea; 2 Department of Radiology, Hallym University College of Medicine, Hallym University Sacred Heart Hospital, Anyang 14068, Republic of Korea; 3 Department of Internal Medicine, Soonchunhyang University College of Medicine, Soonchunhyang University Hospital, Bucheon 14584, Republic of Korea; 4 Department of Surgery, Soonchunhyang University College of Medicine, Soonchunhyang University Hospital, Bucheon 14584, Republic of Korea

**Keywords:** rectal cancer, total neoadjuvant therapy, elective nodal irradiation, lateral pelvic lymph nodes, intensity-modulated radiation therapy, tomotherapy

## Abstract

With the introduction of total neoadjuvant therapy (TNT) in locally advanced rectal cancer treatment, multidisciplinary treatment options have become more diverse than before, and many challenges remain unresolved. A randomized clinical study in intermediate-risk locally advanced rectal cancer showed that neoadjuvant full-dose systemic chemotherapy with response-adapted omission of radiation therapy is non-inferior to concurrent chemoradiotherapy. Given that preoperative systemic chemotherapy provides an additional layer of local disease control, the traditional role and extent of neoadjuvant radiation therapy could be strategically re-evaluated within the TNT framework. In this context, a risk-adapted approach featuring selective reduction in elective nodal irradiation volume, particularly of the lateral pelvic lymph nodes, may offer a promising middle ground for treatment personalization. Drawing parallels from surgical practice—where total mesorectal excision is standard but lateral pelvic lymph node dissection is reserved for selected cases—this perspective advocates for similar selectivity in radiation therapy targeting, focusing on mesorectal and presacral regions while judiciously omitting lateral nodes in appropriately selected patients. This approach could maintain oncologic safety by focusing radiation therapy on limited but essential volumes. With modern intensity-modulated radiation therapy, reducing the target volume translates directly to enhanced organs-at-risk sparing, thereby mitigating radiation-induced toxicity. When combined with induction chemotherapy response assessment to refine patient selection, this approach can offer a biologically informed, personalized treatment paradigm that balances disease control with quality of life.

## 1. Introduction

The treatment landscape of locally advanced rectal cancer (LARC) has evolved substantially over the past two decades. The German Rectal Cancer Study established neoadjuvant chemoradiotherapy (CRT) as the standard of care [1]. Subsequently, the RAPIDO and PRODIGE-23 trials demonstrated that total neoadjuvant therapy (TNT) achieves superior oncologic outcomes compared to conventional CRT [2,3,4]. Additionally, the PROSPECT trial showed that neoadjuvant FOLFOX (folinic acid, fluorouracil, and oxaliplatin) with selective radiation therapy (RT) omission achieves non-inferior disease-free survival to CRT in selected patients [5].

The PROSPECT trial evaluated a dichotomous approach to RT by enrolling 1194 patients with intermediate-risk LARC (T2N+ or T3N0-1) amenable to sphincter-sparing surgery. The intervention arm received six cycles of modified FOLFOX, with restaging after chemotherapy determining the need for subsequent CRT—only 9% required it due to insufficient response. Five-year disease-free survival was equivalent between arms (81% FOLFOX vs. 79% CRT), validating RT omission in carefully stratified cohorts [5]. However, patient-reported outcomes revealed higher rates of anxiety, neuropathy, nausea, and vomiting during neoadjuvant treatment in the FOLFOX arm [6], highlighting the considerable burden of full-dose oxaliplatin-based chemotherapy.

This binary choice—full systemic therapy without RT versus CRT with less intensive chemotherapy—overlooks a potentially optimal middle ground. In the TNT era, where full-dose systemic chemotherapy is administered early, it may be reasonable to consider selective reduction in RT target volume [7]. Specifically, risk-adapted omission of elective nodal irradiation (ENI) to lateral pelvic lymph nodes (LPLN) in selected patients could potentially enable chemotherapy de-escalation while maintaining local control through RT that is limited in volume but focused on essential therapeutic targets. This approach is informed by surgical practice and utilizes modern intensity-modulated RT (IMRT) capabilities to optimize the therapeutic ratio.

## 2. The PROSPECT Dilemma: Trading One Toxicity for Another

The PROSPECT trial demonstrated that 91% of intermediate-risk LARC patients achieved adequate tumor regression with FOLFOX alone, permitting RT omission [5]. However, the trial simultaneously exposed a critical limitation: to achieve comparable efficacy without RT, patients endured six full cycles of modified FOLFOX over 12 weeks.

The toxicity trade-off in the PROSPECT trial highlights the distinct clinical profiles of each approach. During neoadjuvant treatment, severe (≥Grade 3) acute toxicity was markedly higher with FOLFOX (41%) compared to CRT (22.8%)—nearly double [6]. The FOLFOX arm experienced significantly higher rates of neuropathy (a hallmark of oxaliplatin), along with increased anxiety, nausea, vomiting, and fatigue [6]. In patient-reported outcome domains, CRT was significantly superior in 12 out of 14 symptom domains [6,7].

Conversely, the long-term benefits of avoiding pelvic radiation became more apparent over time. At 12 months after surgery, FOLFOX patients reported lower rates of fatigue and better sexual function compared to CRT patients [6]. However, it is important to note that sexual function data were available for only 20–26% of participants, and the trial predominantly enrolled a relatively favorable intermediate-risk population (85.8% cT3 only, with just 14–17% of tumors within 5 cm of the anal verge), raising concerns about overtreatment in this relatively favorable-risk population [7].

The fundamental question becomes: must we choose between acute oxaliplatin toxicity and chronic radiation effects, or is there a middle ground? For the LARC population where both systemic and local control matter, if we can reduce RT volumes while maintaining local control through risk-stratified selective elective nodal irradiation (ENI) omission, we may decrease chemotherapy intensity, thereby minimizing both toxicities.

## 3. Lessons from the Scalpel: Selective LPLN Management in Surgery

The management of LPLN in rectal cancer surgery provides an instructive parallel. Total mesorectal excision (TME) is universally accepted as the oncologic standard, achieving excellent local control rates [8]. However, lateral pelvic lymph node dissection (LPND)—typically targeting internal iliac and obturator stations—remains controversial and selectively applied [9,10].

Japanese surgeons have traditionally performed LPND routinely for low rectal cancer, based on autopsy studies showing LPLN metastasis rates of 8.8% to 34% [9]. In contrast, Western practice favors neoadjuvant CRT with ENI, reserving LPND for selected cases [10]. Recent evidence suggests both approaches may be insufficient alone; persistent enlarged LPLNs after CRT correlate with increased lateral local recurrence rates [11,12].

The emerging consensus favors a selective, risk-adapted approach: combining neoadjuvant therapy with LPND only when oncologic benefit outweighs surgical morbidity [9]. Pretreatment MRI criteria guide this decision—nodes with short-axis diameter ≥ 7 mm warrant more aggressive management [13,14]. Importantly, post-treatment nodal size also predicts risk; nodes shrinking to <5 mm after neoadjuvant therapy are rarely pathologically positive [15].

This surgical philosophy—TME for all, LPND for selected high-risk patients—could inform RT target design. If lateral node dissection is reserved for clearly indicated cases, why routinely irradiate lateral nodes in patients who receive early systemic chemotherapy that may reduce the need for pelvic ENI?

## 4. Rethinking ENI in the TNT Era

Traditional RT target volumes for rectal cancer evolved from analyses of postoperative recurrence patterns, and the latest National Comprehensive Cancer Network guidelines define these volumes to include the mesorectal and presacral regions plus lateral lymphatic stations—specifically the posterior obturator and internal iliac nodes, with external iliac nodes added for T4 tumors involving anterior structures [16,17,18]. This approach made sense in the conventional paradigm, where preoperative CRT, with chemotherapy serving primarily as a radiosensitizer to enhance locoregional tumor control, represented the standard approach before surgery [19,20,21]. However, major trials including the German Rectal Cancer Study and EORTC 22921 found that adjuvant chemotherapy after preoperative CRT did not yield convincing survival improvements [1,22].

The advent of TNT has shifted this calculus. In PRODIGE-23, patients received six cycles of FOLFIRINOX before CRT [3,4]. In RAPIDO, short-course RT was followed by six cycles of CAPOX/FOLFOX [2]. In these paradigms, full-dose systemic chemotherapy addresses micrometastatic disease early—largely what ENI was meant to accomplish.

Recurrence pattern analyses support reconsidering ENI. A study examining 126 patients with local recurrence after neoadjuvant therapy found that 75.4% recurred within the internal pelvic cavity; among 186 recurrent lesions, mesenteric regions accounted for 40.9% and presacral regions 32.3%, whereas the lateral lymphatic drainage region (obturator, internal iliac, and external iliac) comprised only 10.2%, indicating relatively infrequent recurrences there [23]. The authors questioned whether ENI combined with involved-field irradiation to high-risk areas may constitute overtreatment in the TNT era, when robust systemic therapy is administered regardless.

Evidence from mesorectal-limited RT studies demonstrates that excluding ENI of lateral pelvic and superior rectal nodes allows further reduction in organ-at-risk (OAR) doses (small bowel and bladder) compared to conventional field RT [24]. This volume reduction is expected to decrease acute gastrointestinal toxicity.

In the TNT era, it may be reasonable to reserve routine LPLN-ENI for truly high-risk patients, while defining the mesorectum and presacral regions as the standard essential fields.

## 5. Identifying Patients Suitable for Limited-Volume RT

A critical question for implementing this approach is patient selection: which patients have sufficiently low LPLN risk to be candidates for limited-volume RT? Drawing from the PROSPECT trial’s patient population, intermediate-risk LARC (cT2N1 or cT3N0-1, with N1 in central/mesorectal nodes, amenable to sphincter-sparing approaches) represent possible candidates for this approach. Given the limitations of baseline nodal classification, pretreatment staging alone can be insufficient to determine treatment volume selection. Rather, integrated assessment of primary tumor response and post-induction nodal imaging can provide more reliable biological information for treatment decisions [25,26]. While the consolidation versus induction chemotherapy debate continues [27], a robust response to induction chemotherapy suggests chemosensitive biology and the possible sterilization of micrometastatic lateral nodal disease, justifying selective limitation of RT volumes [24].

**Figure 1:**
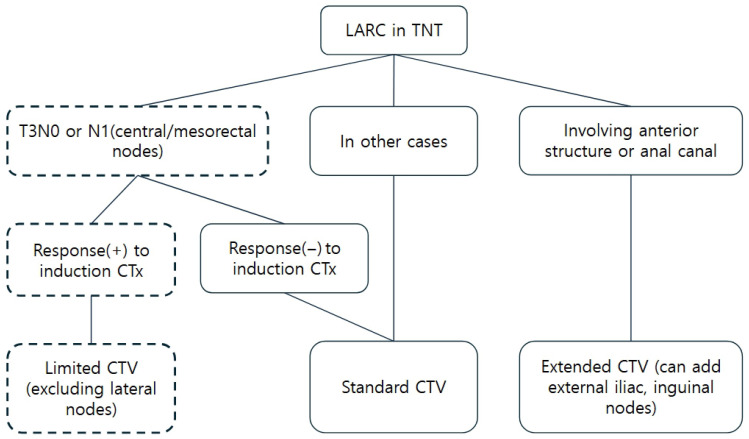
Proposed clinical decision algorithm for risk-adapted RT target volume tailoring in LARC treated with TNT. The dashed box on the left highlights the novel conceptual framework of risk-adapted ENI proposed in this article. CTV = clinical target volume.

In contrast, patients with clearly suspicious LPLN involvement at baseline or extensive T4 disease require comprehensive RT including full lateral ENI. For patients with baseline extramural venous invasion or mesorectal fascia involvement, post-induction reassessment is critical: persistent high-risk features or poor tumor response favor comprehensive ENI, while substantial response with clearance of these features may allow biology-guided limited-volume RT [24]. The risk-stratified populations described above represent an opportunity for biology-guided limited-volume RT in the TNT era, warranting clinical investigation. Figure 1 depicts our proposed selection algorithm and clinical decision framework for managing LARC in the TNT era.

## 6. The IMRT Advantage: Volume Reduction Equals Toxicity Reduction

Radiation oncology has undergone remarkable technological evolution. Initially, two-dimensional treatment planning was based on radiographs of bony pelvic structures to approximate target volumes, often resulting in substantial irradiation of normal tissues. Combining CT-based planning with the use of multileaf collimators facilitated three-dimensional conformal RT (3DCRT), enabling identification and delineation of relevant target volumes in three dimensions. However, the true paradigm shift came with inverse- planned treatment techniques, including IMRT, volumetric modulated arc therapy (VMAT), and helical tomotherapy (a specialized form of IMRT) [28]. In the current era, radiation oncologists precisely delineate target volumes on axial CT images, while simultaneously contouring OAR. By modulating radiation intensity within individual treatment beams, these techniques enable sparing of normal tissue contained between concavely shaped pelvic lymph node targets and allow dose painting to optimize therapeutic ratios [29].

These modern techniques are critical enablers of selective ENI. Unlike 3DCRT, IMRT/VMAT/tomotherapy deliver highly conformal dose distributions with steep dose gradients, allowing superior sparing of OAR. For rectal cancer, the planning target volume (PTV) size directly impacts OAR doses. Dosimetric planning studies demonstrate that mesorectal-limited RT, which excludes LPLN and superior rectal nodes, substantially reduces small bowel and bladder doses compared to conventional approaches [24]. The magnitude of OAR sparing is directly proportional to the volume of elective nodal regions omitted, with each reduction in PTV translating to measurable decreases in OAR dose-volume parameters.

**Figure 2:**
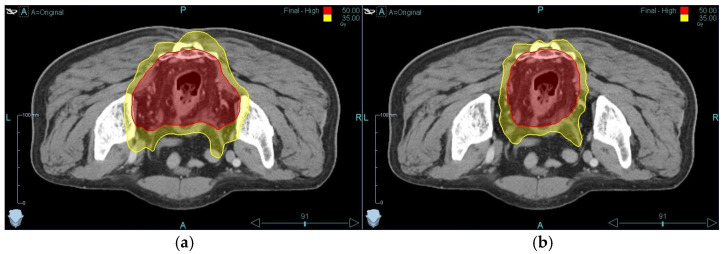
Tomotherapy plan comparison in a representative LARC patient. Isodose lines are shown for (**a**) the conventional PTV and (**b**) the reduced PTV.

A tomotherapy planning comparison in a representative LARC patient illustrated the dosimetric benefits of selective ENI omission (Figure 2). Transitioning from standard pelvic RT (PTV 734.7 cm^3^) to reduced-volume RT targeting mesorectal and presacral regions only (PTV 470.4 cm^3^) achieved a 36.0% PTV reduction. This volume reduction translated to substantial OAR sparing (Table 1): small bowel V45Gy decreased from 61.8 cm^3^ to 30.9 cm^3^, representing a 50.0% reduction—a clinically meaningful reduction as V45Gy is most strongly associated with gastrointestinal toxicity [30]. Femoral head mean dose decreased from 14.6 Gy to 11.8 Gy (19.2% reduction), while bladder mean dose remained stable (9.1 Gy vs. 8.9 Gy). This plan was generated in the prone position on a belly board, displacing the small bowel in a ventro-cranial direction [31], and a supine setup would likely yield additional bowel sparing with limited PTV. While these findings demonstrate proof of concept from a single-patient planning study, larger-scale dosimetric analyses are warranted to systematically quantify OAR sparing benefits of reduced-volume RT using advanced techniques across broader patient populations.

Grade 1–2 acute toxicities—including diarrhea, urgency, proctitis, and fatigue—commonly occur during standard pelvic RT with conventional ENI and can substantially impact quality of life. The substantial small bowel dose reductions achievable through selective ENI omission may decrease the incidence and severity of these toxicities, thereby potentially improving treatment tolerability and patient-reported outcomes. Such dosimetric improvements are likely to manifest clinically as reduced acute diarrhea, lower risk of chronic bowel dysfunction, preserved bladder capacity, and decreased late femoral insufficiency fractures [32].

## 7. Addressing Potential Concerns

A critical question regarding selective omission of lateral ENI is whether this strategy will increase lateral local recurrence rates. Accumulating evidence suggests that selective omission is likely safe in appropriately selected patients. Analysis of recurrence patterns after neoadjuvant therapy reveals that the majority of pelvic recurrences occur within mesorectal and presacral regions, with lateral lymphatic drainage regions accounting for only a minority of failures [23]. Even in challenging cases of low rectal cancer with anal sphincter invasion, omitting inguinal and external iliac irradiation resulted in only 3.7% inguinal and 3.3% external iliac failure rates at 3 years, with truly isolated failures being rare [33].

Regarding imaging accuracy, a legitimate concern is whether MRI can reliably identify LPLN involvement. Modern high-resolution MRI with thin-slice T2 sequences and diffusion-weighted imaging provides good sensitivity for detecting suspicious lymph nodes using combined size and morphologic criteria [11,13]. While false positives and false negatives inevitably occur in imaging-based nodal assessment, using both baseline nodal size and post-induction chemotherapy response enhances diagnostic accuracy. Specifically, the LPLN must demonstrate a short-axis diameter of less than 7 mm on pretreatment MRI and less than 5 mm following induction chemotherapy, along with the absence of suspicious morphological features—including irregular or spiculated borders, heterogeneous internal signal intensity, a round shape, and the loss of the fatty hilum [13,14]. Furthermore, a favorable clinical response can be defined as a primary tumor size decrease of at least 30% according to RECIST 1.1 criteria [34]. Assuming isotropic regression, this 30% reduction in the longest diameter translates to an approximately 66% reduction in tumor volume [25,26], thereby serving as a stringent threshold for identifying favorable responders eligible for this selective de-escalation approach.

Our proposal for selective ENI operationalizes biology-informed de-escalation demonstrated by watch-and-wait (W&W) strategies, and recent evidence shows that even high-risk LARC patients can safely pursue W&W if they achieve complete clinical response to TNT [35,36]. Importantly, this selective ENI could be specifically designed for patients undergoing standard surgery, where surgical resection provides definitive locoregional control; for patients pursuing W&W, RT becomes the sole locoregional treatment, necessitating comprehensive standard-volume coverage to ensure adequate tumor control and minimize the risk of marginal recurrence.

## 8. Limitations and Future Directions

Several limitations of this study warrant consideration. First, as a perspective article, this work is primarily intended to propose a conceptual framework and generate a hypothesis for the TNT era, rather than reporting quantitative clinical outcome data. Consequently, the lack of long-term oncologic evidence regarding the safety of selective ENI omission remains a primary limitation that must be addressed through future prospective trials. Second, the dosimetric support for the proposed de-escalated target volume is derived from a proof-of-concept planning study using a representative case. While this illustrates the technical feasibility of sparing OAR, its results are intended for conceptual visualization and do not offer the statistical power of large-scale dosimetric studies. Third, while we draw a conceptual analogy to the surgical risk-adaptive paradigm, we recognize that surgical LPND (for lateral cN+ disease) and ENI omission (for lateral cN0 disease) are not biologically identical. Consequently, the safety of omitting elective irradiation remains a hypothesis that requires prospective validation.

Future work should integrate emerging molecular and imaging biomarkers to refine patient selection and RT volume decisions. Circulating tumor DNA (ctDNA) clearance during induction chemotherapy can serve as a potent indicator of treatment response. Studies show that colorectal cancer patients whose ctDNA rapidly becomes undetectable after induction therapy have a higher likelihood of pathologic complete response and a lower risk of local recurrence [37]. This suggests that ctDNA monitoring could provide critical supplementary insights to refine risk stratification beyond imaging-based assessment, conceivably helping identify optimal candidates for the selective ENI strategy. Radiomics analysis may serve as a valuable tool for identifying imaging phenotypes that yield complementary quantitative metrics to assess suitability for limited-volume RT. By extracting high-dimensional features (texture, shape, intensity) from MRI/CT, radiomics could potentially refine risk-stratification by capturing subtle biological nuances beyond standard morphological evaluation. Notably, it has been demonstrated that T2-weighted MRI-based radiomics features achieved higher accuracy in predicting pathologic complete response of rectal cancer compared to qualitative assessment by radiologists, further supporting the potential of imaging-derived parameters in personalized treatment planning [38]. As these emerging findings are integrated to enhance the precision of response assessment, the scope of selective target volume tailoring—currently cautiously limited to the relatively favorable-risk subgroup—could potentially be expanded to include higher-risk populations in the future.

MRI-guided adaptive RT techniques can further facilitate the precise implementation of de-escalated target volumes by providing a sophisticated platform for real-time anatomical verification. By continuously imaging the pelvis during the treatment course, these adaptive workflows allow for dynamic plan adjustment in response to tumor shrinkage and organ motion, ensuring that the RT target remains accurately aligned with the residual disease while maximizing the sparing of adjacent normal tissues [39,40]. This capability can offer an additional layer of technical confidence when pursuing a selective ENI strategy.

## 9. Conclusions

Given that preoperative systemic chemotherapy can provide supplementary local disease control, the strategic de-escalation of radiation volumes may be worth exploring within the TNT framework. Our proposed risk-adapted model suggests that selective ENI might serve as a balanced approach to personalization. Inspired by the surgical practice of selective LPND, this perspective proposes that radiation targeting could be similarly refined, focusing on essential volumes while selectively considering the omission of lateral nodes in favorable cases.

Notable gaps remain in current TNT trial design: lack of protocol-mandated RT techniques and incomplete adoption of IMRT across participating centers. Modern IMRT enables precise volume sculpting analogous to surgical resection planes—targeting specific nodal regions tailored to individual disease extent while achieving meaningful toxicity reduction. Future TNT research should move beyond the binary “RT or no RT” framing to incorporate risk-adaptive, personalized RT volume strategies using standardized advanced RT techniques. When combined with induction chemotherapy response assessment, this approach enables treatment intensification for aggressive disease while sparing good responders from unnecessary morbidity, potentially optimizing the balance between oncologic control and quality of life. Selective ENI represents a refined paradigm in precision radiation oncology, facilitating the delivery of optimized treatment volumes tailored to individual risk profiles and biological responses within the rectal cancer TNT framework.

## Figures and Tables

**Table 1:** Dose-volume parameters in conventional vs. limited-volume pelvic RT.

Target or OAR	Parameters	Conventional Target	Limited Target	Reduction (%)
PTV	Volume (cm^3^)	734.7	470.4	36.0
Bowel	V45Gy (cm^3^)	61.8	30.9	50.0
	V40Gy (cm^3^)	80.2	43.3	46.0
	V30Gy (cm^3^)	123.1	75.6	38.6
Bladder	Dmean (Gy)	9.1	8.9	2.2
Femur head	Dmean (Gy)	14.6	11.8	19.2
	Dmax (Gy)	32.6	23.7	27.3

V45Gy = the volume receiving ≥ 45 Gy, Dmean = mean dose, Dmax = maximum dose.

## Data Availability

All data generated or analyzed during this study are included in this article.
